# Supplementary material for: Sensibility and Specificity of the VitaPCR™ SARS-CoV-2 Assay for the Rapid Diagnosis of COVID-19 in Older Adults in the Emergency Department
Source: Viruses. 2023 Jan 9;15(1):189. doi: 10.3390/v15010189 (PMC9866422; doi:10.3390/v15010189)
Supplement: Supplementary file 1 [file viruses-15-00189-s001.zip › viruses-2085066-supplementary.pdf]

Table S1. Comparison between the VitaPCR™ and other molecular RDTs.

Instrument	Ref	Sensitivity	Time (min) for the analysis	Advantages	Drawbacks
ID NOW (Abbott)	[13] [22]	84% (95% CI 55–96%); 85% (95% CI 75%-92%)	5-30	fast time to response, largely used	low sensitivity
Xpert Xpress assay (Cepheid)	[23]	99% (95% CI, 97-99%)	45-50	high sensitivity	large time to response
CovidNudge	[24]	94% (95% CI 86-98%)	90	high sensitivity, high number of genes detected	lack of validation studies; large time to response
VitaPCR (Menarini diagn.)	[17,18]	90% (nd); 99.3 % (nd); 83.4% (95% CI 81.5-85.2)	20	good sensitivity, fast time to response	lack of validation studies
Accula (Mesa Biotech)	[15]	68% (95% CI 58-77%)	30	none	low sensitivity, low specificity

nd: not declare.d
